# Perioperative Management for Port Catheter Procedures in Pediatric Patients with Severe Hemophilia and Inhibitors

**DOI:** 10.1055/a-2337-3687

**Published:** 2024-09-12

**Authors:** Anna Woestemeier, Silvia Horneff, Vincent Marlon Lüder, Jennifer Nadal, Arne Koscielny, Jörg C. Kalff, Johannes Oldenburg, Georg Goldmann, Philipp Lingohr

**Affiliations:** 1 Department for General, Visceral, Thoracic and Vascular Surgery, University Hospital of Bonn, Bonn, Germany; 2 Institute of Experimental Haematology and Transfusions Medicine, University Hospital of Bonn, Bonn, Germany; 3 Institute for Medical Biometrics, Informatics and Epidemiology, University Hospital of Bonn, Bonn, Germany; 4 Department for General and Visceral Surgery, St. Elisabeth-Hospital Leipzig, Leipzig, Germany

**Keywords:** hemophilia A, hemophilia B, port catheter, inhibitors, antibodies, rFVIIa

## Abstract

**Background:**

The objective of this systematic study was to assess the perioperative management and outcome of surgery in pediatric patients with hemophilia A/B and inhibitors compared to nonhemophilic pediatric patients.

**Methods:**

The surgical outcome of 69 port catheter operations in patients with hemophilia who developed inhibitory antibodies against the administered factor was compared to 51 procedures in the control group. In the patients with hemophilia and inhibitors, a standardized protocol for recombinant activated factor VII was used to prevent perioperative bleeding.

**Results:**

Hemophilic pediatric patients with inhibitors showed no significant differences in perioperative management (blood transfusion: *p* = 0.067, duration of surgery: *p* = 0.69; *p* = 0.824) in comparison to patients without hemophilia *.* The length of hospital stay was significantly longer in pediatric patients with hemophilia and inhibitors (20 days vs. 4 days for insertion; 12 days vs. 1 day for explantation). Moreover, no statistically significant difference was found for secondary bleeding (three patients with hemophilia vs. none in the control group; *p* = 0.11) or surgical complications (five hemophilia patients vs. none with grade I complication; one hemophilia patient vs. none with grade II complications; *p* = 0.067).

**Conclusion:**

This study has demonstrated that port catheter insertion and removal is safe in these patients. Moreover, it shows the importance of a coordinated approach with a multidisciplinary team.

## Introduction

Hemophilia A and B are inherited bleeding disorders characterized by a deficiency of clotting factor VIII (FVIII) in hemophilia A and factor IX (FIX) in hemophilia B. Nowadays, prophylactic treatment with clotting factor concentrate is the gold standard of therapy and key to a good long-term outcome. Especially bleeding into the joint is the main cause of chronic pain and disability in these patients. Prophylactic administration of factor concentrate in children with severe hemophilia should start early in childhood before manifestation of the first joint bleed. 1 2 3

However, up to a third of patients with severe hemophilia A and 3% of patients with severe hemophilia B develop inhibitory antibodies against the administered factor that leave the patient at risk for life-threatening bleeding. Inhibitor development usually occurs within the first 50 days of treatment mainly triggered by the type of the underlying mutation. 4 Immune tolerance induction (ITI) with frequent application of high doses of factor concentrate was the treatment of choice, while this study was conducted for patients with inhibitors and is successful in about 70% of patients with hemophilia A 5 and in 25% of patients with hemophilia B. 6 However, recently the humanized bispecific antibody with affinity to FIX/FIXa and FX emicizumab has expanded options to treat hemophilia A. Studies showed that emicizumab prophylaxis was highly effective at preventing bleeding in patients with and without inhibitors and therefore the need for inhibitor eradication has become less certain for patients. 7 8 Especially in very young children, venipuncture is often difficult and traumatic. For ITI with frequent administration of coagulation factors, central venous access devices (CVADs) are necessary to guarantee clotting factor application and to avoid repeated traumatic peripheral venous punctures. 9 10 Furthermore, a safe venous access enables parents to perform home treatment after parent training. Recent studies showed no difference in perioperative complications in adult patients with hemophilia without inhibitors undergoing surgery such as appendectomy, inguinal hernia repair, hemorrhoidectomy, cholecystectomy, and transurethral prostate or bladder surgery. Compared to controls, only the duration of hospital stay was significantly longer. 11 12 13

The objective of this study was to assess the perioperative management and outcome of surgery in children with hemophilia and inhibitors compared to nonhemophilic pediatric patients.

## Materials and Methods

### Study Design and Patients

This retrospective study included a total of 59 consecutive patients who underwent port catheter insertions and/or explantations at the University Hospital Bonn and the Asklepios Children's Hospital St. Augustin, Germany, between 1992 and 2017. Informed consent was obtained from all patients and the study was approved by the Medical Ethics Committee. The surgical outcome of the 69 port catheter operations for ITI in patients with hemophilia who developed inhibitory antibodies against the administered factor was compared to 51 procedures in cancer patients requiring a port catheter insertion for chemotherapy.

Data were collected regarding patient's age, gender, diagnosis and indication for insertion, type of mutation, date of insertion and removal of port catheter, complications, duration of operation, length of hospital stay, and the protocol of peri- and postoperative factor administration.

### Perioperative Clotting Factor Therapy

To prevent perioperative bleeding, the following protocol was applied. Administration of recombinant activated FVII (rFVIIa; 90–100 µg per kg body weight [BW]):

- Day of surgery: 2 hours preoperatively, at incision, then every 2 hours.
- 1st and 2nd postoperative day every 2 hours.
- 3rd and 4th postoperative day every 3 hours.
- 5th and 6th postoperative day every 4 hours.
- 7th and 8th postoperative day every 6 hours.
- 9th and 10th postoperative day every 8 hours.
- 11th and 12th postoperative day every 12 hours.

After completing the protocol, induction of immune tolerance was continued with high doses of FVIII concentrate twice daily 100 IE/kg BW and prothrombin complex concentrate twice daily 50 IE/kg BW.

### Surgical Approach

Pediatric surgeons implanted all the devices under general anesthesia. All patients received antibiotic prophylaxis before insertion. Two techniques were used for catheter insertion: open cut-down technique and percutaneous technique. Sites of insertion included the subclavian, internal jugular, and external jugular veins.

### Statistical Analysis

We performed statistical analysis using SPSS version 25 (IBM Corp., IBM SPSS Statistics, Chicago, Illinois, United States). The continuous variables were presented as means and standard deviation, and categorical variables were presented as numbers and percent. To determine statistical significance, the Mann–Whitney U-test was used. Categorical variables were compared using the chi-square test if the expected frequency was less than 5. A *p* -value of less than 0.05 was considered statistically significant.

## Results

### Patient Characteristics

During the study period, a total of 69 port catheter insertion and removal procedures was performed in 34 hemophilia patients. The median age of patients was 1 year (range: 0–15 years) and the median weight was 10.9 kg (range: 7–46 kg). All patients were male. Patient characteristics are shown in Table 1 .

**Table 1:** Patient characteristics and postoperative outcome

	Hemophilia patients	Control patients	*p* -Value
Number of patients	34	25	
Age (median)	1	12	
Sex			
Male	34	10	
Female	0	15	
Weight (kg)	10.9	38.2	
Procedures (total)	69	51	
First implantation	31	30	
First explantation	14	20	
Second implantation	8		
Second explantation	8		
Change	8	1	
Duration of surgery (min)			
Implantation	40	47	0.824
Explantation	20	20	0.690
Hospital stay (d)			
Implantation	20	4	0.001
Explantation	12	1	0.001
Complications a			
Grade I	5	0	0.067
Grade II	1	0	
Secondary bleeding b	3	0	0.110

a Complications according to the Clavien–Dindo classification.

b Bleeding occurring within 10 days after the operation.

Twenty-four patients (70%) had developed clinically relevant inhibitors (≥0.4 Bethesda units/mL) before catheter insertion. In 18 (75%) of these patients, an Intron 22 inversion was detected in the F8 gene. Intron 22 inversion has a 25 to 30% risk for inhibitor development. 14 The average length of hospitalization was 20 days after insertion and 12 days after removal of port catheter.

In the control group ( *n* = 25), a total of 51 port catheter insertion and removal procedures were performed during the study period. The median age of patients was 12 years (range: 0–17 years) and the median weight was 38.2 kg (10.5–101 kg). Fifteen patients were female and 10 were male.

### Duration of Surgery

The duration of surgery was defined as cut to suture time. In pediatric hemophilic patients, the mean length of operation was 40 minutes (range: 28–140 minutes) for port catheter placement and 20 minutes for its removal. In the control group, the median duration of surgery was 47 minutes (range: 28–82 minutes) for port catheter placement and 20 minutes for its removal. When comparing the two groups, no significant difference was found ( *p* = 0.69; *p* = 0.824).

### Length of Hospital Stay

The length of hospital stay was significantly longer in pediatric patients with hemophilia and inhibitors (20 days for port catheter insertion and 12 days for explantation) compared to the control group (4 days in patients with port catheter placement and 1 day for its removal) ( Table 1 ).

### Blood Loss and Postoperative Complications

**Fig. 1:**
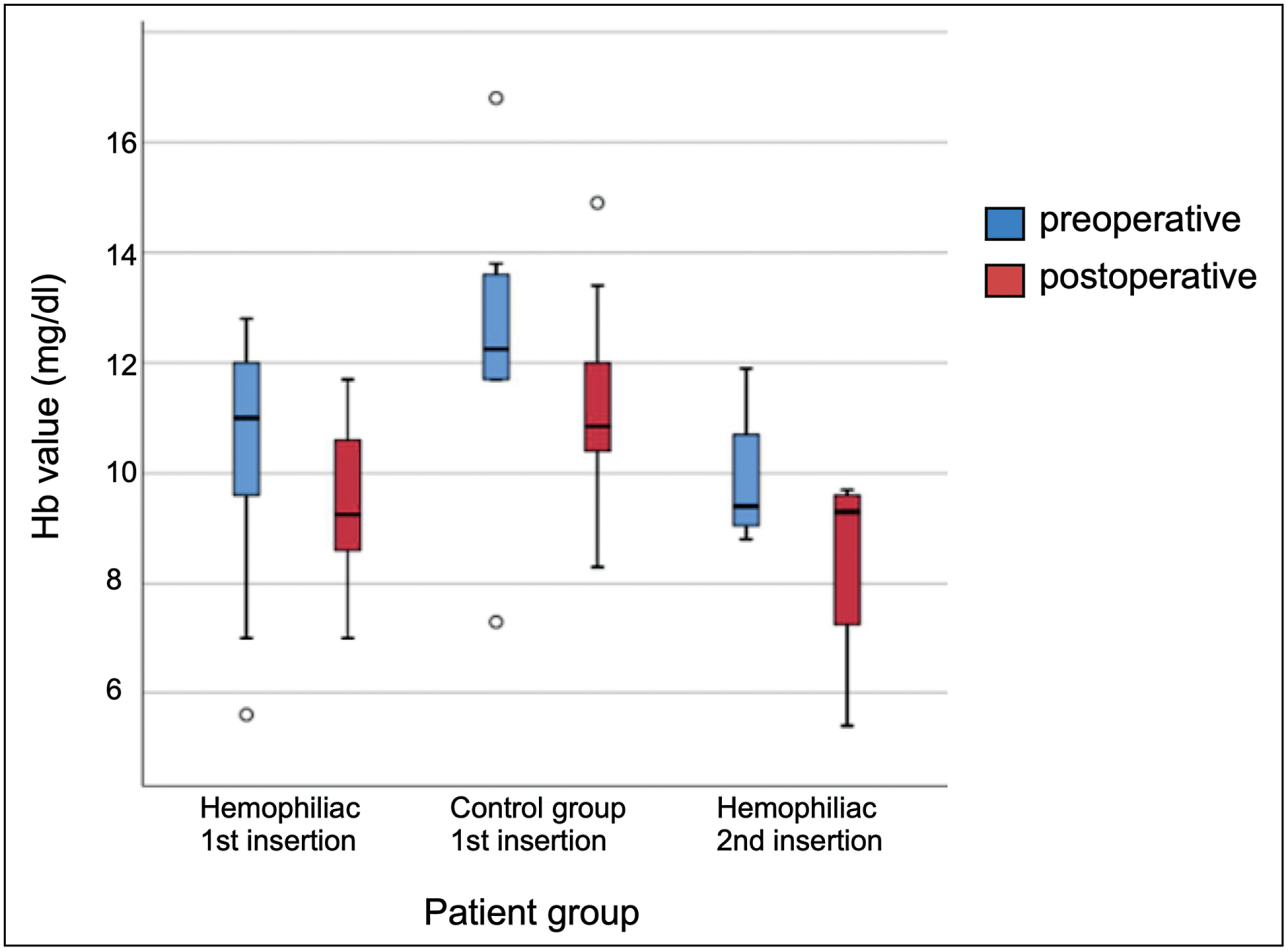
Pre- and postoperative Hb values. Hb, hemoglobin.

The mean preoperative hemoglobin level in pediatric hemophilic patients was 11.2 mg/dL (range: 9–13.1) and 11.1 mg/dL (range: 8.4–12.8) in the control group. The mean postoperative hemoglobin level in pediatric hemophilic patients was 10.5 mg/dL (range: 8.4–12.9) and 11.1 (range: 9.9–12.4) in the control group. The hemoglobin levels did not differ when comparing the two groups ( Fig. 1 ).

The severity of surgical complications was ranked according to the Clavien–Dindo classification. 15 Five patients developed grade I complications, such as a postoperative hematoma without the need for pharmacological treatment or surgical interventions. Postoperatively, one patient needed a blood transfusion and thus was classified as grade II complication. The control group did not develop complications ( Table 1 ). There was a trend in complications in patients with hemophilia and inhibitors, but this was statistically not significant ( *p* = 0.067).

During the port catheter placement, three patients with hemophilia developed a bleeding within 10 days after the operation (classified as secondary bleeding), while no bleeding occurred in the control group. This was statistically not significant ( *p* = 0.11).

## Discussion

Historically, surgical procedures for patients with hemophilia were severely limited due to higher risks of intra- and postoperative bleeding, infections, and transfusion-associated transmission of infectious agents. 16 However, with the availability of purified clotting factor concentrates and improved and standardized protocols, surgery in hemophilic patients became safe. 17 With respect to Port-A-Cath implantations and explantations, there is a significant variability in real-world perioperative management. Therefore, we described our periprocedural management and its outcomes for Port-A-Cath implantations and explantations.

Recent studies for some types of abdominal and urological surgeries showed no significant differences in the length of operation, the need for blood transfusion, and the development of postoperative complications between patients with hemophilia and patients without hemophilia. In our study, the length of hospital stay was significantly longer in pediatric patients with hemophilia and inhibitors, due to further application of clotting factor concentrate for wound healing and training of the parents to use the port catheter at home to perform ITI. This was in line with recently published data. 11 12 18 Intensified training on port catheter handling and factor application, which is even started preoperatively, could shorten the length of hospital stay. The presence of a comprehensive home care infrastructure is also one of the key necessities to shorten the length of hospital stay. Shortening the duration of the protocol for postoperative prophylaxis to make the protocol possible for low- and middle-income bears the risk of bleeding and impaired wound healing.

Due to our periprocedural management, we also found no significant difference in the length of operation when comparing patients with and without hemophilia.

Furthermore, no statistically significant differences in postoperative complications occurred when comparing children with hemophilia to the control group, which is due to an excellent interdisciplinary work of surgeons, nurses, anesthesiologists, and hematologists. In patients with high-titer inhibitors, successful surgery has been reported under hemostatic cover with porcine FVIII, plasma-derived activated prothrombin complex concentrate (pd–aPCC), and rFVIIa. However, the published data are usually case series, mostly in adult patients. 19 20 21 This is a systematic study on hemophilic pediatric patients with inhibitors showing that port catheter insertion and removal is safe in this patients.

In a small case series, O'Connell et al evaluated the use of recombinant factor VIIa (rVIIa) for the treatment of acute bleeding in 12 pediatric patients and concluded that rVIIa therapy is the treatment of choice for the management of surgery and acute life- or limb-threatening bleeding. 20 rVIIa was administered at a dose of 90 μg/kg intravenously 2-hourly for the first 24 hours postoperatively and 4-hourly for the second 24 hours postoperatively and was then stopped in the absence of bleeding in one center (The National Children's Hospital, Dublin). In the other center (Great Ormond Street Hospital, London), the same dose of rVIIa was given 2-hourly for 24 hours, 3-hourly for 24 hours, and 4-hourly for 24 hours. Again, treatment was then discontinued if there was no evidence of bleeding. Our standardized protocol also administered 90 to 100 µg per kg body weight rFVIIa; however over a longer period and shorter intervals (day of surgery: 2 hours preoperatively, at incision, then every 2 hours; 1st and 2nd postoperative day every 2 hours, 3rd and 4th postoperative day every 3 hours, 5th and 6th postoperative day every 4 hours, 7th and 8th postoperative day every 6 hours, 9th and 10th postoperative day every 8 hours, 11th and 12th postoperative day every 12 hours). The more intense regimen was chosen due to clinical observations and experiences in our center. In contrast to factor VIII, bleeding control with bypassing agents is not reliably predictable. Especially in patients with high-titer inhibitors, patients may not respond well to therapy.

A systematic Cochrane review also showed high efficiency rates (>80%) for pd-aPCC in the control of acute bleeding events, with comparable tolerability and low rate of thrombotic complications. 22 Thus, this could be used as an alternative to rFVIIa.

We presented the perioperative management for port catheter insertion and explantation in pediatric patients with hemophilia and inhibitors at the Hemophilia Comprehensive Care Center Bonn. Patients with hemophilia showed no significant differences in perioperative management (blood transfusion, duration of surgery) and postoperative outcome (hemorrhages or other complications) in comparison to patients without hemophilia.

In trend, there were more bleeding complications in patients with hemophilia: five patients developed grade I complications according to the Clavien–Dindo classification, while one patient needed a blood transfusion and was classified as grade II. Nevertheless, this was statistically not significant ( *p* = 0.067).

Ingerslev et al included two pediatric patients in a cohort of patients undergoing major surgery (synovectomy and craniotomy) where rVIIa was successfully used without complications. 23 O'Connell et al described rVIIa to be successful in resolving bleeding in a small series of children, although fibrin glue was needed in one case and red cell transfusion was necessary on two occasions. 20 The reliability and safety of rVIIa in securing perioperative hemostasis was also demonstrated by Shapiro et al. 24 In this randomized trial, two doses of FVIIa (35 vs. 90 μg/kg) were tested for a variety of procedures (including orthopedic procedures, CVAD insertion, and renal biopsies). All high-dose patients (90 μg/kg) and 12/15 low-dose patients (35 μg/kg) had satisfactory hemostasis during the first 48 hours, thus the 35 μg/kg dose was considered sub-optimal for postoperative management.

This study has several limitations, including its retrospective design and the single-center experience in the era prior to the availability of emicizumab. Therefore, the regimen and the results cannot be transferred to patients who are currently treated with emicizumab. The phase IIIb multicenter, single-arm STASEY study evaluated the safety and tolerability of emicizumab prophylaxis in people with hemophilia A aged ≥12 years with FVIII inhibitors. 25 The data support that emicizumab prophylaxis can provide hemostatic coverage during minor and major surgeries, with appropriate concomitant prophylactic hemostatic medication when required, which is in accordance with other studies. 25 26 However, guidelines for management of surgeries in patients with hemophilia A with/without FVIII inhibitors receiving emicizumab are still missing.

Another weakness of this study is the control group, which consists of older pediatric cancer patients compared to our patients with hemophilia. Cancer can cause a prothrombotic or hypercoagulable state through an altered balance between the coagulation and fibrinolytic factors. Pediatric patients with cancer hold an increased risk of venous thromboembolism that is further increased by insertion of central venous catheters. This also might explain the differences in bleeding complications in patients with hemophilia and in patients with cancer in our study.

In conclusion, or study has demonstrated that port catheter insertion and removal is safe in pediatric patients with hemophilia that developed inhibitors. Moreover, it shows the importance of a coordinated approach with a multidisciplinary team of hematologists and surgeons.

## Notes

**What Is Known about This Topic?**

- In patients with high-titer inhibitors, successful surgery has been reported under hemostatic cover with porcine FVIII, plasma-derived activated prothrombin complex concentrate (pd–aPCC), and recombinant activated FVII (rFVIIa). However, the published data are usually case series, mostly in adult patients.
- For Port-A-Cath implantations and explantations, there is a significant variability in real-world perioperative management.

**What Does This Paper Add?**

- This is a systematic study on hemophilic pediatric patients with inhibitors showing that port catheter insertion and removal is safe in these patients: no significant differences were found in perioperative management (blood transfusion, duration of surgery) and postoperative outcome (hemorrhages or other complications) in comparison to patients without hemophilia.
- A standardized protocol for surgical procedures in hemophilic pediatric patients with inhibitors.
